# Zebrafish: an efficient vertebrate model for understanding role of gut microbiota

**DOI:** 10.1186/s10020-022-00579-1

**Published:** 2022-12-23

**Authors:** Hui Xia, Huimin Chen, Xue Cheng, Mingzhu Yin, Xiaowei Yao, Jun Ma, Mengzhen Huang, Gang Chen, Hongtao Liu

**Affiliations:** 1 College of Basic Medicine, Hubei University of Chinese Medicine, Huangjiahu West Road 16, Hongshan Disctrict, Wuhan, 430065 China; 2 Hubei Provincial Hospital of Traditional Chinese Medicine, Wuhan, 430061 China

**Keywords:** Gut microbiota, Zebrafish, Germ-free, Host-microbe interaction, Disease

## Abstract

Gut microbiota plays a critical role in the maintenance of host health. As a low-cost and genetically tractable vertebrate model, zebrafish have been widely used for biological research. Zebrafish and humans share some similarities in intestinal physiology and function, and this allows zebrafish to be a surrogate model for investigating the crosstalk between the gut microbiota and host. Especially, zebrafish have features such as high fecundity, external fertilization, and early optical transparency. These enable the researchers to employ the fish to address questions not easily addressed in other animal models. In this review, we described the intestine structure of zebrafish. Also, we summarized the methods of generating a gnotobiotic zebrafish model, the factors affecting its intestinal flora, and the study progress of gut microbiota functions in zebrafish. Finally, we discussed the limitations and challenges of the zebrafish model for gut microbiota studies. In summary, this review established that zebrafish is an attractive research tool to understand mechanistic insights into host-microbe interaction.

## Introduction

As an emerging research field, microbiomics has developed rapidly in the past decade because of the advent of sequencing technologies, which can reveal the taxonomic identity and functional traits of microorganisms without cultivation. Furthermore, the significance of host-associated microbiota in health maintenance has been successfully revealed in humans and other animal models. There is growing evidence that dysbiosis or unfavorable changes in the microbiome are closely associated with various human diseases, such as inflammatory bowel disease, obesity, diabetes mellitus, cardiovascular disease, and cancer (Lozupone et al. 2012; Sommer and Bäckhed 2013; Lescak and Milligan-Myhre 2017). However, the investigation into their causal relationships is constrained due to ethical issues. For this reason, several animal models have been successfully developed for microbiome studies, like mice, rats, *Drosophila melanogaster*, *Caenorhabditis elegans*, and zebrafish (*Danio rerio*) (Douglas 2019).

Zebrafish is favored in life science research due to its small size, high fecundity, early optical transparency, rapid external development, manageable laboratory husbandry, and available genomic resources. So far, zebrafish have emerged as a widely used vertebrate model in biological research fields, including reproduction, development, toxicology, nutrition, and genetics (Phillips and Westerfield 2014; Meyers 2018; Zhang et al. 2020; Zhong et al. 2022). Below we describe the benefits of using zebrafish to understand molecular and cellular mechanisms linked to host-microbe interactions. First, the optical transparency of zebrafish larvae and the availability of transgenic lines expressing fluorescent proteins in distinct cell lineages allow a high-resolution in vivo imaging of intestinal physiology and microbial dynamics at the larval stage of development (Rawls et al. 2007; Hall et al. 2008; Wiles et al. 2020; Schlomann and Parthasarathy 2021). Second, given that gut functions and immune genes are relatively conserved between zebrafish and mammals (Lickwar et al. 2017; Brugman 2016), genetic manipulation approaches applied in zebrafish have propelled us to understand the effect of host gene expression on gut microbiota. Third, large numbers of zebrafish can be cultured in a shared and easily sampled aquatic environment, which makes the large degree of biological replication in zebrafish possible to explore the extensive inter-individual variation observed in vertebrate-associated microbiota (Rogers et al. 2014; Stephens et al. 2016). Besides, it is easy to generate germ-free (GF) zebrafish embryos by surface sterilization of the chorion, thus allowing convenient control of their microbial environment (Pham et al. 2008; Melancon et al. 2017). Finally, it is relatively easy to manipulate zebrafish with specific genes or microbiomes. For instance, researchers can explore specific functions of intestinal microorganisms by introducing a specific microorganism in GF zebrafish larvae (Stagaman et al. 2020). These advantages mentioned above make zebrafish an effective tool to exploit the effect of intestinal bacteria on host health. This review presents zebrafish as an efficient vertebrate model to study the host-microbe interaction in humans. We aim to get insight into the critical role of the zebrafish model in gut microbiota research, which may benefit knowledge of the complex intestinal environment in mammals.

## Intestinal structure of zebrafish

The intestinal development in larval zebrafish occurs in three main stages. Stage I is featured by lumen formation and the conversion of a thin ribbon of endoderm into a tubular structure from 26 h post-fertilization (hpf) to 52 hpf, which is the development stage from hatching to the opening of the mouth in zebrafish. Stage II is characterized by the proliferation and polarization of intestinal epithelial cells before 76 hpf, which is the development stage from the mouth opening to the anus opening. Stage III is the development stage from the anus opening to the complete resorption of yolk (76–126 hpf), featured by the remodeling and differentiation of intestinal epithelium, which means that the majority of the digestive system is functional in larval zebrafish at stage III (Wallace and Pack 2003; Ng et al. 2005). However, it will take another four weeks to achieve the proliferation pattern of adult fish. At 6 to 19 days post fertilization (dpf), the intestine grows in length at a lower proliferation rate, which is restricted to the epithelial interfold base. Between 20 to 33 dpf, the intestine undergoes metamorphosis with deepened epithelial folds and intestinal looping. And the proliferative progeny moves up with an eventual maturation of the intestinal epithelium into the adult structure (Li et al. 2020; Wallace et al. 2005). Therefore, the adult intestine has a more complex structure with a loop that folds back on itself to create an S shape compared with the larval intestine.

The intestines of zebrafish and mammals are highly homologous in their development, structural composition, and biological function. In addition, the distribution of antimicrobial gene expression and leukocyte distribution along the anterior–posterior axis of the intestine is relatively conserved between zebrafish and mammals (Oehlers et al. 2011). Adult zebrafish intestines can be divided into seven segments (S1 to S7) of equal length from the anterior to the posterior region, based on transcriptome results (Wang et al. 2010). Given the different heights of villar ridges in segments S1-S7, the intestine is further regrouped into three morphologically distinct segments S1–S5, S6, and S7. The small intestine gene markers include villin (*vil1*), fatty acid-binding protein 2 (*fabp2*), apolipoprotein 1(*apoa1*), and apolipoprotein 4 (*apoa4*). Their expression patterns suggest that segments S1-S4 are analogous to the mammalian small intestine and closely related to the absorption of protein and lipids. Segment S5 forms a transitional region. Segments S6 and S7 have molecular features of the mammalian large intestine with increased expression of aquaporin 3 (*aq3p*) and cofilin1 (*cfl1*), which are separately involved in the water absorption and dynamic stabilization of actin filaments (Wang et al. 2010). Based on genome-wide profiling of mRNA levels and chromatin accessibility, Lickwar et al. show that the gene expression levels and regulatory regions of intestinal epithelial cells (IECs) are conserved in vertebrates. Many aspects of IECs physiology are also conserved since the common ancestor of zebrafish and mammals (Lickwar et al. 2017). In mammals, bile salts are modified by intestinal microbiota and influence signaling through the bile salt-binding transcription factor farnesoid X receptor (Fxr). Wen et al. show that there is evolutionary conservation of key components of the bile salt-Fxr signaling axis between zebrafish and mammals. The cellular differentiation and physiological programs deployed in the anterior intestine of zebrafish are specialized for bile salt recovery, similar to the mammalian ileum (Wen et al. 2021). Importantly, the highly endocytic vacuolated cells, named lysosome-rich enterocytes (LREs), are found in the ileum of suckling mammals and the mid-intestine of zebrafish. Zebrafish LREs share the same morphology, marker expression, and activity as mice. However, unlike mammals, where these cells are restricted to the neonatal stage, LREs are still retained in adult zebrafish. That might be because zebrafish have no stomach, making their luminal environment similar to suckling mammals. Thus, zebrafish still require vacuolated enterocytes for protein absorption and digestion during adulthood (Wallace et al. 2005). Studies show that LREs preferentially internalize dietary proteins via fluid-phase and receptor-mediated endocytosis and digest them intracellularly both in the immature gut of zebrafish and suckling mice. Thus, these data indicate a highly conserved cellular mechanism of protein absorption between mammals and zebrafish (Park et al. 2019).

**Fig. 1:**
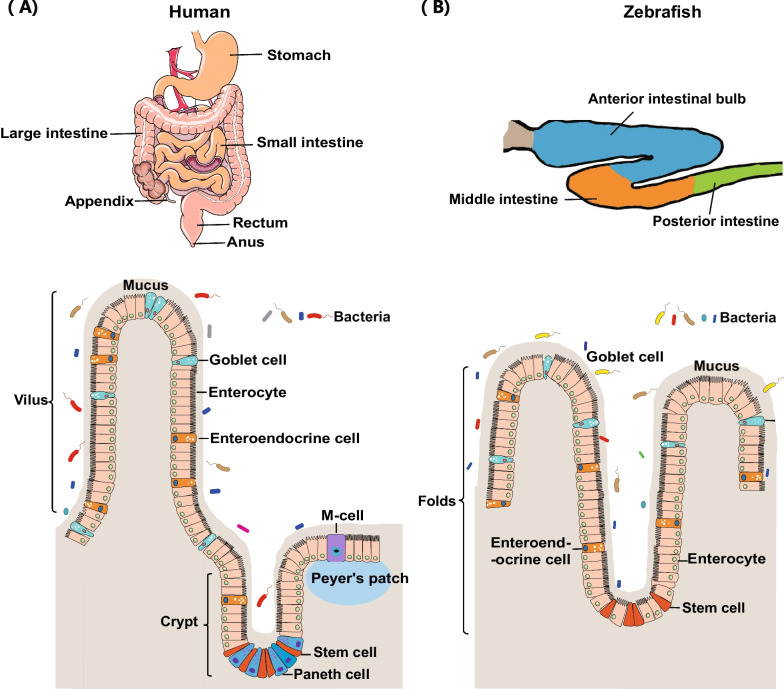
Abdominal anatomy of intestinal tracts between humans **A** and adult zebrafish (**B**). Epithelial cells are specified as either secretory or enterocytes as they migrate up the villi in mammals or folds in zebrafish. The mature zebrafish intestine has three segments: anterior intestinal bulb (blue), middle intestine (orange), and posterior intestine (green). Mature epithelial cells include enterocyte, enteroendocrine, and goblet cells. Compared with the human intestine, crypts, Paneth cells, and M-cells are absent in the zebrafish intestine

Although the intestines of zebrafish and mammals are highly homologous in their development, structural composition, and biological function, there are typical architectural differences in digestive systems (Fig. 1) (Flores et al. 2020; Carten and Farber 2009). In mammals, the gastrointestinal tract is an extremely long and convoluted tube comprising four distinct parts: esophagus, stomach, small intestine (duodenum, jejunum, and ileum), and colon. However, adult zebrafish have no stomach, and their intestine is a simple tapered tube folded into three segments: anterior intestinal bulb, middle intestine, and posterior intestine. The intestine of zebrafish performs essential physiological functions, such as digestion, nutrient absorption, and metabolic homeostasis. Instead of a stomach, the anterior intestinal bulb of zebrafish has a bigger lumen than the posterior part and thus may function as a reservoir comparable to the mammalian stomach. Moreover, the anterior intestine of zebrafish is not acidified for the lack of gastric glands, which serve as a regulatory cue for gastrointestinal pathogens in humans (Ramos-Morales 2012).

Given that the composition of the enteric nervous system and the intestinal architecture in zebrafish are highly conserved as compared to those of mammals at genomic and functional levels (Phelps et al. 2017; Ferguson and Foley 2021), zebrafish have emerged as a valuable model for intestinal diseases such as inflammatory bowel diseases (Oehlers et al. 2017) and Hirschsprung disease (Hamilton et al. 2022; Rolig et al. 2017; Wiles et al. 2016). Histological analyses show that the zebrafish intestine wall comprises three layers: mucosa (consisting of the epithelium and lamina propria), muscularis externa (circular and longitudinal smooth muscle), and serosa layer. Zebrafish intestinal epithelium is organized into irregular folds that protrude into the intestinal lumen and increase its surface area for better nutrient absorption. These folds resemble the villi of the mammalian small intestine, and they are similar in cross-section (Wallace et al. 2005). Zebrafish have no Paneth cells and Peyer’s patches (Fig. 1) and lack submucosa layer and muscularis mucosa (Wallace et al. 2005; Wang et al. 2010; Flores et al. 2020; Ferguson and Foley 2021; Lobert et al. 2016). Hence, their enterocytes, enteroendocrine cells, and goblet cells are present with conserved functions in nutrient absorption, hormone production, and mucus secretion. Zebrafish digestive enzymes are strongly expressed in the anterior segment, where the folds are the longest. Several enzymes are functionally equivalent to mammalian gastric markers like rennin, lipase, and cathepsins (Wang et al. 2010; Flores et al. 2020). And more notably, the vertebrate intestine is innervated not only by the intrinsic enteric nervous system but also by extrinsic efferent and afferent nerves (Uesaka et al. 2016). Similar to mammals, the tryptophan catabolites of intestinal bacteria can activate Trpa1 channels in enteroendocrine cells and stimulate 5-HT secretion, causing the activation of enteric and vagal neurons in zebrafish (Ye et al. 2021). In brief, highly conserved gene expression and biological functions in the gut between zebrafish and mammals suggest the potential of zebrafish as an alternative model in investigating gut microbiota.

## Generation of gnotobiotic zebrafish model

Gnotobiotic animal models are usually used to understand the role of intestinal microbes in their interactions with the host. Remarkably, the advantages of optical transparency in embryos and easy genetic manipulation make GF zebrafish an attractive organism to explore the function of gut microbiota and host-microbiome crosstalk. Because of their *ex utero* fertilization, zebrafish embryos can be surface sterilized and raised without microorganisms. In 2004, Rawls established the protocol for generating a germ-free zebrafish model (Rawls et al. 2004). Now researchers can rear zebrafish under GF or gnotobiotic conditions, which makes it possible to rigorously control the microbial environment in zebrafish (Pham et al. 2008; Melancon et al. 2017). Applying GF zebrafish as a convenient animal model has opened a new era in intestinal microbiome research.

**Fig. 2:**
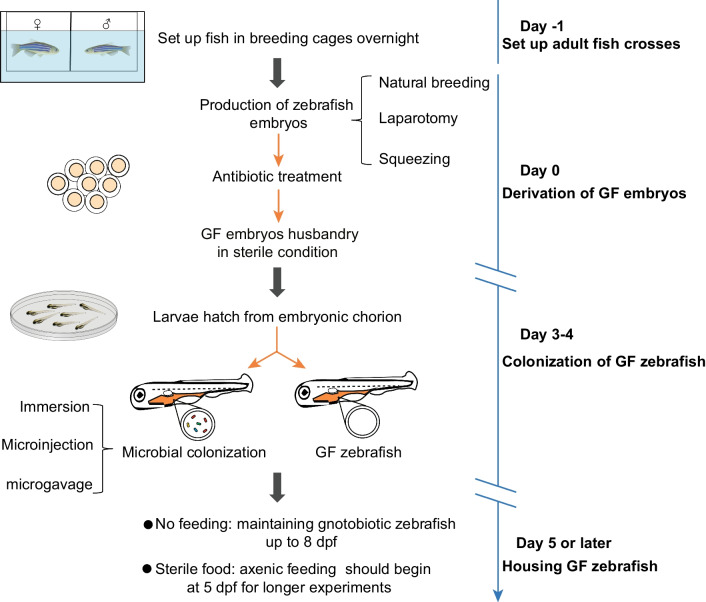
Overview of generation procedures for germ-free zebrafish. On day -1, pairs of adult zebrafish are placed in breeding cages overnight, with males and females separated using a divider. The next morning (Day 0), embryos are collected, rinsed, and transferred to a petri dish containing a filter-sterilized embryo medium. Three general methods are used to obtain gnotobiotic zebrafish embryos, including natural breeding, laparotomy, and squeezing. After the larvae are transferred into sterile culture flasks or gnotobiotic isolator, they are maintained in an incubator to develop at 28.5 °C. On 4 dpf, GF zebrafish colonized by microbiota

Based on reports (Pham et al. 2008; Melancon et al. 2017), we summarize the critical procedure for GF zebrafish generation (Fig. 2). There are three general methods to obtain gnotobiotic zebrafish embryos, including natural breeding, laparotomy, and squeezing. Natural breeding has a higher fertilization rate, fewer technical demands, and minimized damage to the breeding adults, but a higher risk of microbial load. In contrast, the other two methods have low microbial burdens, whereas they are time-consuming and require higher technical requirements. Once the mouth and anus open at about 4 dpf, zebrafish larvae are subsequently permissive to microbial colonization. Therefore, the appropriate colonization time is approximately 4 dpf (Pham et al. 2008; Melancon et al. 2017). To ensure that zebrafish remains free of exogenous microbes, researchers need to monitor sterility every few days during the experiment. Visual inspection can be used as a preliminary screening to detect microbial contamination, so sterility monitoring is usually conducted with phase optics before feeding and water changes (Melancon et al. 2017). For short-term experiments, GF zebrafish need no feeding and can be kept in culture flasks up to 8 dpf until the yolk is almost completely reabsorbed. However, sterile food is still required at 5 dpf for long-term GF zebrafish husbandry.

Experimental approaches for studying intestinal biology often require the in vivo introduction of selected microbes into gnotobiotic zebrafish. Depending on different practical purposes, researchers can add the intestinal contents of conventionally-raised zebrafish to GF zebrafish for microbial colonization. Alternatively, GF zebrafish can be inoculated with microbes present in the media from tanks housing conventionally-raised zebrafish. Rather than colonizing GF fish with complex microbiota, it is more rigorous to colonize fish with individual microbial species to generate mono-associated zebrafish (Rolig et al. 2015). The common colonization methods include immersion, microinjection, or microgavage (Zhang et al. 2020). It is convenient to colonize GF zebrafish by immersing larvae in culture media with microorganisms (Pham et al. 2008). Still, immersion exposure may cause unintended toxicity on extraintestinal tissues, and it is difficult to control the time and route of microbe colonization. The microinjection method involves euthanizing zebrafish larvae, and inoculating microbe directly into the gut lumen via the anal pore or the body wall using a capillary needle (Toh et al. 2013). However, this method is hard to operate and may cause tissue damage to zebrafish. Microgavage can be performed with the help of a microinjector and stereomicroscope, and gavage needles are utilized to inject loading microbes into the mouth of individual zebrafish (Cocchiaro and Rawls 2013; Robinson et al. 2018). Although microinjection and microgavage are labor-intensive, they allow the intestinal lumen to load high contents of microbes, which is lethal to zebrafish with immersion exposure. Also, both methods guarantee the precise delivery of microbes to hosts without escaping into the medium (Cocchiaro and Rawls 2013). However, it should be noted that microinjection or microgavage hasn’t been used currently to precisely control the zebrafish gut microbial composition in the gnotobiotic settings, because larvae are no longer germ-free after the procedure. After that the 16S rRNA amplicon sequencing technique is performed to identify and compare bacteria present within a given sample. Generally, it requires a 0.3 g sample for 16S rRNA amplicon sequencing.

Given that zebrafish have smaller intestines and fewer intestinal contents, multiple zebrafish (approximately 12–20 zebrafish larvae or 6–10 adult zebrafish intestinal contents in each sample) need to be mixed for 16S rRNA amplicon sequencing analysis (Wang et al. 2021; Davis et al. 2016a; Zhang et al. 2019; Zhao et al. 1987). Although the pretreatment method differs from mammals, a larger sample number is more valuable for investigating complex microbial communities. Further, the variation of resident microbial communities among zebrafish samples has little difference in the same tanks, which is beneficial in illustrating the consistency of microbial taxa present within a given group of zebrafish. Therefore, zebrafish can achieve more remarkable statistical ability for microbiota composition analysis.

Compared to gnotobiotic mice, the generation of GF zebrafish larvae is more cost-effective, time-efficient, and high-yield. More importantly, the optical transparency of zebrafish larvae allows the in vivo visualization of intestinal physiology and microbial dynamics in living individuals by light-sheet fluorescence microscopy (LSFM) (Booth and Smith 2020). Parthasarathy and coworkers developed a new optical and computational approach to monitoring the dynamics of specific microbes in the intestinal tract. They found that the composition of the microbial community was affected by several factors, such as host-microbe interactions, microbial population dynamics, and higher-order crosstalk (Wiles et al. 2016; Jemielita et al. 2014; Parthasarathy 2018; Sundarraman et al. 2020). The application of LSFM to transparent zebrafish larvae unveiled unprecedented insights into the interaction between living hosts and their gut microbiota. Until now, the GF zebrafish model has been widely used to study the effect of microbial colonization or metabolism on host physiology like intestinal epithelial maturation (Bates et al. 2006; Cheesman et al. 2011), nutrient metabolism (Semova et al. 2012; Manuneedhi Cholan et al. 2022), immune regulation (Bates et al. 2007; Kanther et al. 2011; Koch et al. 2018), and behavior development (Phelps et al. 2017; Davis et al. 2016a).

## Gut microbiota in zebrafish

### Composition of gut microbiota in zebrafish

**Fig. 3:**
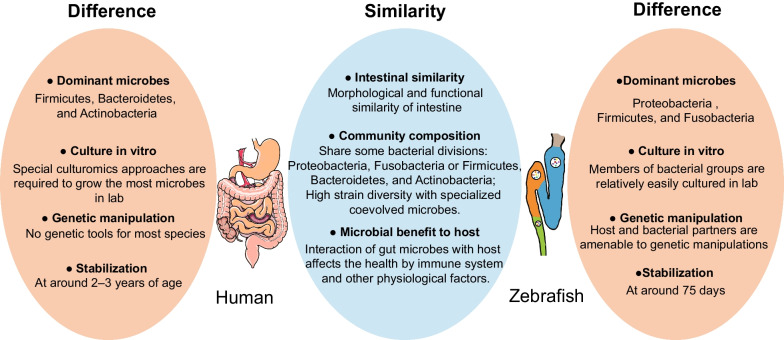
Physiological function comparison of gut microbiota between zebrafish and humans

The gut microbial compositions of zebrafish and mammals share six bacterial divisions at the phylum level, including Proteobacteria, Fusobacteria or Firmicutes, Bacteroidetes, Actinobacteria, Verrucomicrobia phyla (Rawls et al. 2006). These microbes have similar metabolic functions (Fig. 3). However, the human gastrointestinal tract is primarily dominated by Firmicutes, Bacteroidetes, and Actinobacteria at the phylum level, while zebrafish is dominated by members of the phylum Proteobacteria (*Aeromonas*, *Pseudomona*s, and *Vibrio* genera), followed by Firmicutes or Fusobacteria (Flores et al. 2020; Eckburg et al. 2005). With the development of zebrafish, the diversity of the gut microbiota at the phylum level is significantly changed at larval, juvenile, and adult stages, particularly in the composition of Proteobacteria. Although the *γ*-proteobacteria is the most abundant class of bacteria during all development stages, zebrafish intestines show a marked increase of α-proteobacteria at the juvenile stage (21dpf) and are subsequently expanded to *β*-proteobacteria in 35 dpf. However, the proportion of these Proteobacteria classes decreased remarkably in adult zebrafish (75 dpf) (Stephens et al. 2016). By 16S rRNA gene amplicon sequencing, it is demonstrated that members of the phylum Proteobacteria dominate zebrafish gut microbiota at all developmental time points. At the later adult stage, there will be an expansion of members in the phyla Firmicutes and Fusobacteria (Kanther and Rawls 2010; Murdoch and Rawls 2019). Although the dominant phyla are different from those of humans, zebrafish gut microbiota induces relatively conserved host responses during colonization and development, as will be discussed in “Functional studies on gut microbiota using zebrafish model” section.

### Influencing factors of gut microbiota in zebrafish

A series of studies suggest that gut microbiota diversities are affected by extrinsic or intrinsic factors specific to individual species, and gut microbiota in the hosts display day-to-day fluctuations and individual variations (Butt and Volkoff 2019; Yukgehnaish et al. 2020; Patula et al. 2021; Breen et al. 2019; Li et al. 2022a).

#### Extrinsic factors

In most cases, the composition and colonization of gut microbiota in zebrafish can be affected by different extrinsic factors (Zhang et al. 2020; Robinson et al. 2018; Marques et al. 2006; Cornuault et al. 2021). The most studied extrinsic factors include aquatic environment pollutants, diet, antibiotics, and pathogens. As an aquatic animal species, zebrafish gut microbiome is closely related to the ambient environment. The gut microbiota of zebrafish is significantly changed in different aquatic environments, and the gut bacterial community structure varies among domesticated adult zebrafish in geographically separate lab aquaculture facilities (Roeselers et al. 2011). Meanwhile, the α and β-diversity of zebrafish intestinal microbiome change immediately after switching the housing systems (Breen et al. 2019). Besides, environmental pollutants present in the water environment can also alter the composition of intestinal microbiota in hosts. For instance, adult zebrafish display the disturbance of immunity-related intestinal microbes after polystyrene microparticles exposure (Jin et al. 1987), and various bisphenol A (BPA)-derivative chemicals lead to the composition changes of gut microbiota in juvenile zebrafish in a dose-dependent manner (Catron et al. 2019). Similarly, triclosan exposure disrupts the structure and ecological dynamics of the intestinal microbiota of adult zebrafish, and such toxic effects can be attenuated by *Lactobacillus plantarum* feeding (Gaulke et al. 2016; Zang et al. 2019). Beyond that, the presence of other water pollutants also shifts the composition of the gut microbiota community in zebrafish, such as disinfection by-products (DBPs), Benzo[a]pyrene (BaP), and brominated polybrominated diphenyl ethers (PBDEs) (Xie et al. 2020; Xue et al. 2019; Chen et al. 1987).

Diet is a potent force shaping the microbial structure in the gut, which in turn affects a range of metabolic, hormonal, and neurological processes. Overall, diversities of bacterial communities vary with the changing diets. In mammals, diet impacts the physiology and development of hosts directly or indirectly by altering the gut microbiota, which also occurs in zebrafish (Emani et al. 2013; Kashyap et al. 2013; Libao-Mercado et al. 2009). Dietary fat is often associated with the altered human and mouse gut microbiome (David et al. 2014; Zhang et al. 2012). In zebrafish, dietary fat density also has a remarkable impact on the composition of intestinal microbes. Probiotic feeding could attenuate weight gain and decrease cholesterol and triglyceride contents in high-fat diet-fed fish (Wong et al. 2015; Falcinelli et al. 2017). Besides, high-fat feeding also alters the gut microbiota community, such as the enrichment of *Acinetobacter* bacteria, a strain sufficient to induce enteroendocrine cell silencing (Ye et al. 2019). In zebrafish, the administration of dietary nucleotides as functional supplements leads to the modification of intestinal microbes and the reduction in fatty acid oxidation, which is correlated with the suppression of microbial overgrowth in the intestine (Guo et al. 2017). Zebrafish can consume natural diets such as algae and vascular plant tissue, which are potential sources of carbohydrates and could be fermented into short-chain fatty acids (SCFAs) by microbiota (Spence et al. 2007; Cholan et al. 2020). In return, SCFAs can modulate the composition of the gut microbiome. Dietary supplementation with SCFAs can regulate gut microbiota, increase immune response and enhance antioxidative capacity in the zebrafish, which protects the host against Aeromonas hydrophila (Li et al. 2022b). Besides, SCFAs can regulate the dysbiosis of microbiota caused by 2,4,6-trinitrobenzene sulfonic acid (TNBS) exposure (Morales Fénero et al. 2021).

Antibiotics have been used to prevent and treat diseases caused by bacterial infection, particularly to treat severe or life-threatening conditions such as pneumonia and sepsis in humans (Svensson et al. 2015; Kollef and Betthauser 2019; Waele and Dhaese 2019). After exposure to a high dose of vancomycin, adult zebrafish have bacterial populations dominated by Fusobacteria, which reduces the severity of enterocolitis (Brugman et al. 2009). Some antibiotics are also used as veterinary medicine or feed supplements to promote the growth healthy of livestock (Carvalho and Santos 2016). Nevertheless, various antibiotic residues are widely detected in aquatic environments because of their high water-solubility, low bioavailability, and widespread misuse. Environmental pollution by low concentrations of antibiotics in the water results in dysbiosis of gut microbiota in zebrafish, potentially reducing resistance to pathogen infections (Zhou et al. 2018b; Kayani et al. 1987). For example, long-term exposure to oxytetracycline, a broad-spectrum antibiotic, induces the alteration of intestinal bacteria, indicative of the increased abundance of Alpha proteobacteria and Actinobacteria in zebrafish adults (Almeida et al. 2019a, b). Nevertheless, Zhou et al. claim that exposure to oxytetracycline and sulfamethoxazole decreases intestinal microbial richness and increases the abundance of some pathogens, such as *Enterococcus* and *Aeromonas* in adult zebrafish (Zhou et al. 2018a). It is also demonstrated that exposure of zebrafish to environmental concentrations of antibiotics during early life stages increases the abundance of harmful bacteria and antibiotic resistance genes, as well as the metabolic potential of zebrafish gut microbiome (Kayani et al. 1987). Furthermore, the antibiotic treatment leads to a decrease in intestinal microbial diversity of adult zebrafish (Ding and He 2010; Pindling et al. 2018). Nevertheless, using an immersion-based saponin immune stimulation model, researchers have found that oxytetracycline can influence saponin-induced microbiome changes by increasing the *α*-diversity in zebrafish larvae (López Nadal et al. 2018). In another study, as a broad-spectrum antibiotic, the low-dose ciprofloxacin treatment in the aquatic environment dramatically alters gut communities in larval zebrafish, related to the enhanced bacterial aggregation and increased intestinal expulsion (Schlomann et al. 2019). Given all that, antibiotics should be a critical extrinsic factor in manipulating gut microbiota in zebrafish by suppressing the growth of specific bacteria.

Colonization of the host by pathogens can be an extrinsically driven event that could induce alterations to the gut microbiota of zebrafish. The introduction of the aquatic pathogen *Aeromonas hydrophila* disrupts gut homeostasis by inhibiting beneficial bacteria and increasing detrimental bacteria, leading to innate immune responses in the intestine of zebrafish (Yang et al. 2017). Similarly, infection with an intestinal nematode parasite *Pseudocapillaria tomentosa* disrupts the gut microbiome composition of zebrafish (Gaulke et al. 2019). *Vibrio cholerae*, an intestinal pathogen, colonizes the intestine of zebrafish and alters the microbial composition, and induces the type six secretion systems (T6SS) that are pivotal for the pathogenesis (Breen et al. 2021a, b; Logan et al. 2018). Given the complex responses caused by pathogens when colonizing the host, further research is needed to understand the interactions between the pathogen and gut microbiota.

#### Intrinsic factors

Several intrinsic factors (e.g. genetics, age, health, and sex) influence the gut microbiota of zebrafish (Yukgehnaish et al. 2020). The host factor is a critical reason for the difference in intestinal bacteria between zebrafish and other species. Upon transplantation of mouse intestinal microbes (dominated by Firmicutes and Bacteroidetes phyla) into zebrafish, the relative abundance of Proteobacteria phylum increases toward the microbial composition of zebrafish. Vice-versa, when the intestinal bacteria from zebrafish (dominated by the Proteobacteria phylum) are transplanted to GF mouse recipients, the disadvantaged Bacteroidetes and Firmicutes phyla grow out to become dominant (Rawls et al. 2006; López Nadal et al. 2020). Hence, the gut microbiota is under selective pressure within the intestinal habitat of a specific host, and gut microbiota can also influence the host in turn.

The microbial composition undergoes dynamic changes during host aging, and the effect of gut microbiota on health has particular relevance for older people (O'Toole and Jeffery 2015). Before 75 dpf, the microbial community composition changes over zebrafish development (Stephens et al. 2016; Cantas et al. 2012). Cantas et al. report that zebrafish juveniles have a higher bacterial richness in their intestinal microbes compared to the later stages (Cantas et al. 2012). Similarly, zebrafish microbiomes changed in both composition and diversity across the lifespan, as displayed by the reduced α-diversity and raised β-diversity throughout the development (Stephens et al. 2016). Wong et al. also provide evidence that gut microbiota is associated with developmental stages without alterations in diet composition (Wong et al. 2015). Notably, a recent study shows that the development stage is the main factor influencing the microbial composition of zebrafish (Xiao et al. 2021).

It is well-known that host health contributes to a balanced microbial community in the dynamic intestinal environment. Zebrafish and humans share certain similarities in their innate and adaptive immune systems. In early life (until 4–6 weeks post-fertilization), larval zebrafish solely depend on the innate immune system to resist microbial invasion for the lack of adaptive immunity (Flores et al. 2020; Lam et al. 2004). The transparency of embryos has made zebrafish an ideal animal model to elucidate the influence of immune systems on the gut microbiota community. As a part of the gut environment, the immune system plays a key role in shaping the zebrafish microbiomes. By using the zebrafish mutants lacking either innate or adaptive immunity, researchers uncover the potential of the immune system to impose selective pressure on the gut microbiota. Interferon regulatory factor 8 (irf8) is a critical determinant for neutrophil versus macrophage fate choice in zebrafish. Adult *irf8* -deficient zebrafish displays a reduced number of macrophages and severe gut microbiota dysbiosis (an aberrant expansion of Deltaproteobacteria at the expense of Alphaproteobacteria, Gammaproteobacteria, and Fusobacteria), suggesting that the gut macrophages of zebrafish can shape the microbiota by *irf8* (Earley et al. 2018). Recombination activation gene-1 (Rag1) mediates genomic rearrangement and is essential for adaptive immunity in vertebrates. Compared to *rag1*^−/−^ mutant zebrafish, the β-diversity of the intestinal microbiota in wild-type zebrafish individuals is higher, indicating that the intestinal microbes of mutant zebrafish are more homogeneous than those of wild-type individuals (Stagaman et al. 2017). By utilizing *sox10* and *ret* zebrafish mutants associated with Hirschsprung disease in humans, researchers demonstrate that the enteric nervous system can influence microbial community members to promote intestinal health (Rolig et al. 2017; Wiles et al. 2016; Ganz et al. 2018).

Although sex is also one of the factors involved in regulating gut microbiota in mammals (Xie et al. 2017; Bolnick et al. 2014; Zhang et al. 2021a), whether biological sex affects the gut microbial composition of zebrafish is uncertain. Studies report that the composition and diversity of the bacterial community in adult female zebrafish are different from adult males due to the interaction of diet and sex variables (Navarro-Barrón et al. 2019). Silver nanoparticle exposure shows a sex-dependent effect on the richness and diversity of gut microbiota in adult male zebrafish but not in adult females, which is attributed to the fact that male and female zebrafish have different gut bacterial communities (Ma et al. 2018). On the contrary, some studies have reported that the gut microbiota of zebrafish has no significant sex-related differences. However, exposure to endocrine-disrupting agents Estradiol (E2) and BPA can disturb the gut microbial composition in zebrafish, which may be related to changes in host lipid metabolism (Li et al. 2022a; Cantas et al. 2012; Liu et al. 2016). There are differences in the microbiota composition between juvenile and sexually mature zebrafish. Zebrafish juveniles have higher bacterial richness in their gut microbiota than adults, which may result from higher levels of sex hormones in adult zebrafish (Cantas et al. 2012). Recent research shows that male zebrafish have a higher abundance of families (*Erythrobacteraceae* and *Lamiaceae*,) and genera levels (*Lamia* and *Altererythrobacter*) than females. Still, there are no statistical differences in α and β diversity between male and female microbiome composition in phyla, class, and order levels (Martyniuk et al. 2022). The conflicting conclusion may stem from the different aquatic environments in other laboratories or endocrine disruption such as sex hormones. Therefore, further studies should be performed to explore whether or by what mechanisms sex causes the difference in the gut microbiota of zebrafish. In conclusion, these studies reveal the importance of intrinsic factors that affect the intestinal microbes of zebrafish.

## Functional studies on gut microbiota using zebrafish model

In recent years several studies have described the molecular mechanisms behind the host-microbiome interactions using the gnotobiotic zebrafish model, including development, reproduction, immune system, metabolism, and behavior. In this section, we summarize the role of gut microbiota in zebrafish (Table 1) and provide a critical overview of these findings for a complete understanding of this animal model.

**Table 1:** Biological function of gut microbiota in zebrafish

Biological function	Intestinal bacteria	Molecular mechanism	Reference
Development	*Aeromonas veronii* biovar sobria, *Pseudomonas fluorescens*	Promoting intestinal epithelial maturation through distinct host recognition pathways	Bates et al. 2006
	Gut microbiota	Determining the fate of intestinal epithelial secretory cells through Myd88-dependent inhibition of Notch signaling	Troll et al. 2018
	*Aeromonas veronii* HM21	Enhancing the stability of β-catenin in intestinal epithelial cells and promoting cell proliferation in the development of vertebrate intestine	Cheesman et al. 2011
	*Aeromonas veronii* HM21, *Shewanella*	Releasing BefA protein that causes the multiplication of pancreatic β cells	Hill et al. 2016
	*Aeromonas veronii*: dTomato HM21, *Vibrio cholerae:*GFP ZWU0020	Being required for normal neurobehavioral development in early life of zebrafish	Phelps et al. 2017
	*Vibrio*	Excessive growth of *Vibrio* species inhibit the development of adaptive immunity in larval zebrafish	Brugman et al. 2014
Reproduction	*Lactobacillus rhamnosus* IMC 501	Promoting gonadal differentiation through the regulation of GnRH and IGF signalings	Avella et al. 2012; Carnevali et al. 2013
	*Lactobacillus rhamnosus* IMC 501	Regulating ovary physiology by inhibiting follicular apoptosis and increasing follicular survival	Gioacchini et al. 2013)
	*Lactobacillus rhamnosus* IMC 501	Promoting follicle maturation and fecundity by modulating the gene expression of neuropeptide hormones and metabolic signals	Gioacchini et al. 2010; Gioacchini et al. 2011, 2013
	*Pediococcus acidilactici*	As a probiotic supplement in male zebrafish diet for the improvement of molecular parameters in testicular cells	Valcarce et al. 1887
	The commercial probiotic mixture SLAb51	Counteracting the adverse effects of BPA on zebrafish reproduction by interacting with gametogenesis-related genes	Giommi et al. 2021
Immunity	*Aeromonas veronii* biovar sobria, *Pseudomonas fluorescens*, *Streptococcus*, and *Staphylococcus*	Influencing host immunity through the regulation of Myd88 and TNF receptor	Bates et al. 2007
	*Exiguobacterium* ZWU0009*, Chryseobacterium* ZOR0023	Inducing changes in intestinal leukocyte subsets and Myd88-dependent gene expression of host	Koch et al. 2018
	Gut microbiota	Induces expressions of proinflammatory and antiviral factors, which results in the increased resistance of larvae to viral infection	Galindo-Villegas et al. 2012
	Gut microbiota	Suppressing systemic neutrophil activation by microbiota-induced Serum amyloid A	Murdoch et al. 2019
	*Pseudomonas aeruginosa* PAK	Regulating dynamic temporal and spatial transcription activation of NF-кB and subsequent up-regulation of target genes in intestine	Kanther et al. 2011
	*Lactobacillus*	Adhesive probiotic Lactobacillus has anti-infective in protecting zebrafish against pathogenic infections, which is correlated with their spatial distribution in the intestine	He et al. 2017
	*Chryseobacterium massiliae*	Protecting zebrafish from intestinal damage upon *Flavobacterium columnare* infection	Stressmann et al. 2021
Metabolism	*Aeromonas hydrophila* (ATCC 35654)*, Pseudomonas aeruginosa* PA01	Modulating cholesterol metabolism and trafficking through the regulation of *ApoB* and *Fdps*	Rawls et al. 2004
	Gut microbiota	Promoting the uptake of macromolecular proteins in the distal intestine	Bates et al. 2006
	*Exiguobacterium sp. ZWU0009, Chryseobacterium sp. ZOR0023, Pseudomonas sp.*ZWU0006	Stimulating fatty acids uptake and lipid droplet accumulation in intestinal epithelia and liver	Semova et al. 2012
	Gut microbiota	Regulating expression of intestinal Angptl4 and storage of peripheral fat by suppressing the intestine-specific transcriptional enhancer	Camp et al. 2012
	Gut microbiota	Increasing lipid accumulation in intestinal epithelia by regulating gene expression related to lipid metabolism	Sheng et al. 2018
	*Stenotrophomonas maltophilia*, *Enterococcus faecalis*	The mouse high fat-diet-associated microbiota accelerate hyperlipidaemic phenotype in zebrafish embryos	Manuneedhi Cholan et al. 2022
	SCFAs	SCFAs produced by zebrafish microbiota reduces liver phosphoenolpyruvate carboxykinase 1 expression and overall glucose level	Han 2018
Behavior	*Aeromonas veronii:* dTomato HM21, *Vibrio cholerae:*GFP ZWU0020	Being necessary for locomotor activity of zebrafish larvae	Phelps et al. 2017
	*Lactobacillus plantarum*	Modulating communication with CNS via microbiome-gut-brain axis	Davis et al. 2016a, b; Borrelli et al. 2016

### Role of gut microbiota in development

In vertebrates, the most populous and complex microbiota community resides in the gastrointestinal tract, which is essential for host development (Ramos-Morales 2012). Since zebrafish is an excellent model for developmental biology, it is natural that early microbial studies also focus on the larval stage related to zebrafish development (Rawls et al. 2004). Previous studies show that the resident microbes promote the intestinal development of zebrafish, such as epithelial differentiation and proliferation (Bates et al. 2006; Cheesman et al. 2011; Jia et al. 1987). In the absence of microbiota, the intestinal tract of zebrafish displays incomplete development and impaired function that can be reversed by bacterial transplantation, indicating the significance of gut microbiota in intestinal epithelial cell fate and gut development (Bates et al. 2006). Precise regulation of signal transduction pathways is crucial for normal intestinal development and homeostasis in vertebrates. A key β-catenin protein modulates canonical Wnt signaling through its role in cell–cell adhesion complexes. The abundant β-catenin protein accumulates in the cytoplasm and then translocates into the nucleus, where it interacts with coactivators to turn on the transcription of pro-proliferative target genes. Cytoplasmic destruction complex can destroy β-catenin by the proteasome in the absence of endogenous Wnt ligands (Xu et al. 2020). In zebrafish, intestinal microbiota enhances Wnt pathway activity and elevates rates of epithelial cell proliferation in the developing intestine (Cheesman et al. 2011). Troll et al. provide the first evidence that gut microbiota promotes the proliferation of epithelial secretory cells through Myd88 (myeloid differentiation primary response gene 88)-dependent inhibition of Notch signaling in the intestine of zebrafish (Troll et al. 2018). Furthermore, microbial modification of bile salts can modulate farnesoid X receptor (Fxr) activity in zebrafish, which plays a role in repressing genes important for LRE functions in the ileum and promoting genes involved in enterocyte differentiation in the anterior intestine (Wen et al. 2021).

In addition to affecting intestine development, the gut microbiota has a role in pancreatic development. The intestinal microbiota is required for pancreatic β cell expansion during early larval development, and specific bacterial members can restore normal β cell numbers and release β Cell Expansion Factor A (BefA) protein to induce β cells proliferation (Hill et al. 2016). Moreover, growing evidence has proven the role of gut microbiota in neurologic development (Carabotti et al. 2015). In zebrafish, microbial colonization during early life is indispensable for normal neurological development. Microbial disruption with antibiotics alters the neurodevelopment and behavior in larval zebrafish, but the potential mechanisms are unclear (Phelps et al. 2017). These data show that the colonization and composition of gut microbiota during early life are required for zebrafish development.

### Role of gut microbiota in reproduction

Reproduction is an energy-costly process and is sensitive to energy homeostasis.. Several studies find that gut microbiota contributes to the development of gonads and subsequent host reproduction (Aydın et al. 2019). Avella et al. report that the diet supplement with probiotic *Lactobacillus rhamnosus* IMC 501® accelerates gonad differentiation and alters sex ratios in the final population through the regulatory effect on GnRH and IGF systems (Avella et al. 2012; Carnevali et al. 2013). As a feed additive, *L. rhamnosus* promotes follicle maturation, fecundity, and egg quality (Gioacchini et al. 2010a, b; Gioacchini 2011). Besides, low levels of apoptotic signals are essential to maintain homeostasis in reproductive cells and to regulate ovarian function in zebrafish (Thomé et al. 2012), while *L. rhamnosus* IMC 501 treatment can regulate zebrafish ovarian function by inhibiting follicular apoptosis and increasing follicular survival (Gioacchini et al. 2013). Valcarce et al. confirm that the *Pediococcus acidilactici* can be used as a probiotic supplement in the male zebrafish diet to improve reproductive performance (Valcarce et al. 1887). Besides, administration of a commercial probiotic mixture SLAb51 can counteract the adverse effects of BPA on zebrafish reproduction by interacting with gametogenesis-related genes (Giommi et al. 2021). In other studies, the central role of gut microbiota in affecting reproductive efficiency has been demonstrated in mammals (Hussain et al. 2021). Gut microbiota can interact with sex hormones to regulate the reproductive endocrine system. The imbalance of gut microbiota composition can lead to human reproductive pathologies (Kunc et al. 2016; Flores et al. 2012; Qi et al. 2021). Noticeably, although some of the above probiotics like *L. rhamnosus* display beneficial effects on zebrafish reproduction, it is not the indigenous commensal microbiota in the zebrafish intestine. Thus, we should be prudent in extending the conclusions from these bacteria to zebrafish. In the future, more studies will be carried out to identify the role of indigenous commensal bacteria in regulating zebrafish sexual maturity and downstream reproduction events.

### Role of gut microbiota in immunity

There is growing evidence that gut microbiota is crucial for the development and maturation of the immune system, which further mediates a variety of host immune functions (López Nadal et al. 2020). This can be demonstrated by the reduced expression of immune effector genes and increased susceptibility to microbial pathogens in GF animal models in comparison with conventional animals. Moreover, the host immune responses vary with the intestinal microbial composition in mammals (Rooks and Garrett 2016; Belkaid and Harrison 2017). Despite the similarity between humans and zebrafish in immune systems, as discussed in other reviews (Kanther and Rawls 2010; Vliegenthart et al. 2014), zebrafish larvae have no functional adaptive immune system and mostly rely on the innate immune system during the first 2–3 weeks of embryonic development. Hence, zebrafish larvae appear as the effective model for understanding the function of innate immunity when exposed to different bacterial pathogens (Pont and Blanc-Potard 2021). For example, zebrafish goblet cells produce mucus to protect intestinal epithelia when encountering pathogens (Martins et al. 2019). Zebrafish intestinal microbiota can induce intestinal alkaline phosphatase activity, which can dephosphorylate and detoxify the endotoxin component of lipopolysaccharide to regulate inflammatory responses (Bates et al. 2006). MyD88 is the canonical adaptor for inflammatory signaling pathways downstream of members of the Toll-like receptors (TLRs), which act as pattern recognition receptors (PRR) for pathogen-specific molecular patterns (Deguine and Barton 2014). TLRs signaling through MyD88 promotes nuclear translocation of NF-κB and transcription of proinflammatory cytokines such as tumor necrosis factor (TNF). Intestinal microbes affect the host immune state by activating Myd88 and TNF receptors (Bates et al. 2007; Koch et al. 2018). Galindo-Villegas et al. validate commensal microbe recognition is regulated mainly through the TLR/MyD88 signaling pathway. The colonization by commensals in newly hatched zebrafish increases neutrophil recruitment. It induces several genes encoding inflammatory-related factors (IL-1β, TNFα, IL-8, and IL-8–like 2) and antiviral factors (*MxB* and *MxC*), increasing the resistance of larvae to viral infection (Galindo-Villegas et al. 2012). As a biomarker of inflammation, serum amyloid A (SAA) produced by IECs following microbiota colonization promotes local recruitment of neutrophils to the intestine while reducing expression of pro-inflammatory markers and bactericidal activity, thereby allowing neutrophils to effectively respond to other microbes or respond to injury (Murdoch et al. 2019). Single microbial species can also influence the immune system in larval zebrafish. Kanther et al. colonize GF transgenic zebrafish with *Pseudomonas aeruginosa*, which can cause the dynamic temporal and spatial patterns of NF-кB (nuclear factor kappa light chain enhancer of activated B cells) transcriptional activation and leads to the up-regulation of its target genes in the intestinal tract (Kanther et al. 2011). Using the gnotobiotic zebrafish model, Rolig et al. identify an immunomodulatory protein, AimA (Aeromonas immune modulator), and it is secreted by *Aeromonas* and structurally similar to the mammalian immune-modulatory protein lipocalin-2, which is a secreted glycoprotein and obstructs the siderophore iron-acquiring strategy of bacteria to exert immunomodulatory functions in various diseases (Rolig et al. 2018; Xiao et al. 2017). Moreover, AimA could protect *Aeromonas veronii* from the host immune response and thus enhance its intestinal colonization (Rolig et al. 2018).

In addition, researchers also focus on the association between gut microbiomes and host health, particularly the SCFAs produced by microbiota through the anaerobic fermentation of indigestible carbohydrates. Intestinal microbial metabolites SCFAs have a conserved anti-inflammatory effect in zebrafish. Cholan et al. demonstrate that gut commensals from adult zebrafish intestines are capable of synthesizing SCFA in vitro, and butyrate could reduce the recruitment of neutrophils and M1-type to the wound to enhance the anti-inflammatory ability of zebrafish (Cholan et al. 2020). A strain of *Pediococcus pentosaceus* isolated from fish gut enhances the resistance of zebrafish against *Aeromonas hydrophila* by increasing the abundance of SCFA-produced bacteria, butyrate levels, and the expression of IL-1β (Shan et al. 2021). Furthermore, *Escherichia coli* could utilize glucose and produce acidic byproducts in the zebrafish gut, which inhibits the colonization rate of the classical and EI Tor biotypes of *V. cholerae* to prevent or treat cholera infection (Nag et al. 2018).

### Role of gut microbiota in metabolism

The microbial community densely colonizes the intestinal tract, a crucial absorptive organ that contributes a lot to the harvest of dietary nutrients (Sonnenburg et al. 2005; Costello et al. 2009). Currently, a variety of studies have proved the significance of gut microbiota in regulating host metabolism, including fermenting indigestible dietary polysaccharides in humans (Flint et al. 2012; Zhang et al. 2014) and affecting nutrient absorption and energy balance in zebrafish (Semova et al. 2012). Moreover, different intestinal microbial phenotypes are associated with developing different metabolic syndrome symptoms in humans (Kau et al. 2011). The gut microbiota may modulate cholesterol metabolism and trafficking by regulating the levels of Apolipoprotein B (*ApoB*) and farnesyl-diphosphate synthetase (*Fdps*) in zebrafish, as reported by Rawls (Rawls et al. 2004). GF zebrafish decreases protein macromolecule uptake and alters gut motility, all of which can be reversed by colonizing intestinal microbiota, suggesting the ability of the microbiota to regulate digestive functions (Bates et al. 2006). Taking advantage of embryonic transparency and the availability of various transgenic lines, researchers have utilized GF zebrafish to facilitate the knowledge of gut microbiota in nutrient metabolism (Zhang et al. 2020). For instance, Semova et al. prove that the colonization of gut microbiota stimulates fatty acid uptake and lipid droplet accumulation in the intestinal epithelia and liver of zebrafish. Further, diet-enriched Firmicutes increase the number of epithelial lipid droplets, whereas other bacterial types increase the lipid droplet size (Semova et al. 2012). Angiopoietin-like 4 (*Angptl4*) gene in zebrafish is specifically suppressed in intestinal epithelia upon colonization with normal microbiota, providing insight into how gut microbiota influences fat storage (Camp et al. 2012). Cholan et al. identify two specific members of the mouse HFD-associated microbiota *Stenotrophomonas maltophilia* and *Enterococcus faecalis*. They find zebrafish larvae are more susceptible to hyperlipidaemia when exposed to high-fat-fed mouse microbiota (Manuneedhi Cholan et al. 2022). The dietary fat level significantly affects gut microbiota β-diversity at 35 dpf and 70 dpf. A high-fat diet accelerates the establishment of characteristic bacteria in adult zebrafish, such as *Fusobacteria* (Wong et al. 2012). In addition, the expression of genes associated with lipid catabolism and cholesterol synthesis is also affected by intestinal bacteria in zebrafish, suggesting that gut microbiota regulates energy homeostasis (Sheng et al. 2018).

In mammals, the gut microbiota plays a vital role in dietary carbohydrate metabolism (Rowland et al. 2018). SCFAs, such as acetate, propionate and butyrate, are generated by bacterial fermentation of poorly absorbed carbohydrates. SCFAs can increase insulin sensitivity and promote glucose homeostasis in mammals by facilitating the secretion of incretin glucagon-like peptide-1 and binding to the SCFA receptors FFAR2 and FFAR3 on enteroendocrine L-cells (Ducastel et al. 2020; Tolhurst et al. 2012; Portincasa et al. 2022). Similarly, the gut microbiota is involved in the glucose metabolism of zebrafish. BefA proteins produced by genera *Aeromonas* and *Shewanella* could promote pancreatic β-cell proliferation, thereby increasing insulin levels and regulating blood glucose homeostasis (Hill et al. 2016). Besides, the increased abundance of intestinal resident bacteria *Cetobacterium somerae*, an acetate producer, and sodium acetate treatment improve zebrafish glucose homeostasis through parasympathetic activation (Wang et al. 2021). Furthermore, the administration of probiotic *Lactobacillus rhamnosus* can hinder blood glucose elevation by down-regulating the proinflammatory cytokines in the type 2 diabetes mellitus zebrafish model (Bootorabi et al. 2021). Bacterial communities from adult zebrafish intestines synthesize all three main SCFAs in vitro. Propionate treatment reduces liver phosphoenolpyruvate carboxykinase 1 expression and overall glucose level in zebrafish larvae, suggesting SCFAs play an important role in regulating glucose homeostasis (Han 2018).

Given the substantial number of metabolism-associated genes shared with humans, the zebrafish model has been extensively employed to investigate the relationship between metabolic diseases and the gut microbiome. These studies provide new insight into the development of probiotics that can regulate the accumulation of SCFAs and intestinal microbiota homeostasis in zebrafish, indicating that SCFAs can be regarded as a potential immunostimulant against pathogens or drug treatment for metabolic diseases. With the increasing application of the gnotobiotic zebrafish model, the molecular mechanisms of gut microbiota during nutrient absorption can be explored deeply in future investigations.

### Role of gut microbiota in behavior

The microbiota plays a vital role in maintaining host behavior by modulating brain function and altering fundamental behavior patterns. Microbiota dysbiosis can change the function of central and peripheral nervous systems that alters the host’s brain signaling and behavior. In mammalian studies, the aberrant changes in host-associated microbiota are linked to various neuropsychiatric conditions featured with behavioral symptoms, such as Parkinson’s disease, anxiety, and depression (Pirbaglou et al. 2016; Keshavarzian et al. 2015). Zebrafish also exhibit numerous behaviors that could be correlated with those appearing in human neurological processes and disorders (Rico et al. 2011; Lee et al. 2021). Nowadays, zebrafish have emerged as a valuable species to explore the molecular mechanisms underlying a variety of behaviors, including swimming, social communication, sex, cognition, and anxiety (Fetcho et al. 2008; Kalueff et al. 2013). Behavioral changes caused by microbes usually are achieved by microbiota-gut-brain axis communication (Mohanta et al. 2020). The embryonic transparency allows monitoring of the brain’s live development and activity in zebrafish, thus making zebrafish an ideal model to investigate the host-microbe interactions altering behavior (Cornuault et al. 2021). Combined with genetically modified bacteria (Stephens et al. 2015; Wiles et al. 2018), researchers can generate a new transgenic zebrafish model to visualize the morphology or activity of specific neuronal subpopulations (Melancon et al. 2017; Stewart et al. 2014). Therefore, it is necessary to develop zebrafish models of neurobehavioral diseases based on the gut-microbiome-brain axis to deepen human cognition in neurobehavioral diseases.

In zebrafish, the intestinal microbes affect stress responses and behaviors, in particular, anxiety-like and locomotor behaviors, which might, in turn, regulate the feeding behavior and energy homeostasis (Butt and Volkoff 2019). To study the effect of gut microbiota on adult behavior, researchers introduce exogenous bacteria, such as human probiotic strains *L. plantarum* and *L. rhamnosus*, into adult zebrafish. Davis et al. find that the zebrafish larvae supplemented with *L. plantarum* displaye a relieved anxiety-like behavior. In contrast, the characteristic response to an acute stressor is eliminated in gnotobiotic larvae (Davis et al. 2016a). The attenuation of anxiety-related behavior by *L. plantarum* is accompanied by the alteration of GABAergic and serotonergic signaling in the brain. Also, *L. rhamnosus* prevents gut microbiota from stress-induced dysbiosis (Davis et al. 2016b). In addition, *Lactobacillus rhamnosus* IMC 501 administration alters the zebrafish social and explorative behavior by remodeling DNA methylation of behavior-related genes, demonstrating that changes in microbiota composition may affect the host epigenetic landscape and behavior (Borrelli et al. 2016; Cuomo et al. 2021). In addition, exposure to different classes of antibiotics (Chlortetracycline, Ciprofloxacin, and Ceftazidime), adult zebrafish exhibit hyperlocomotion, cognitive decline, and exacerbated aggressive behavior, which is possibly linked to the diversity of symbiotic bacteria (Petersen et al. 2021). Compared to conventionalized and conventionally colonized zebrafish, GF larvae display hyperactivity. Still, colonization of axenic embryos with individual bacterial species *Aeromonas veronii* or *Vibrio cholerae* is sufficient to block locomotor hyperactivity, indicating that normal neurobehavioral development requires microbial colonization in zebrafish (Phelps et al. 2017). Melatonin regulates disorders of neurotransmitter secretion and metabolism of the gut microbiome induced by caffeine via the gut-microbiome-brain axis, which is achieved by increasing the SCFAs production and accelerating the restoration of neurotransmitter secretion levels (Zhang et al. 2021b). These results highlight the importance of gut microbiota in regulating host behavior and neurobehavioral diseases via the gut-microbiome-brain axis. The zebrafish model can be used to screen potential probiotics in treating neurobehavioral disorders. It is believed that the integration of manipulating the intestinal microbiome and in vivo imaging of zebrafish holds great potential for clarifying the contribution of gut microbiota to host behaviors (Stagaman et al. 2020). Next, more work should be done to determine the translatability of these findings to humans.

## Limitations of zebrafish model to gut microbiota study

As a low-cost, genetically tractable vertebrate species with high physiological and genetic homology to humans, zebrafish have become a promising model organism for gut microbiota study. However, zebrafish is an aquatic organism that shows differences from humans, especially in diets and environmental conditions. Hence there are some limitations to the use of zebrafish for gut microbiota studies. (1) Due to the limited knowledge of zebrafish stage-specific nutritional requirements, most studies involving axenic zebrafish are restricted to young larvae for the lack of appropriate sterile food to sustain GF zebrafish into adult stages (Zhang et al. 2020; Pham et al. 2008; Ferguson and Foley 2021). Therefore, more efforts should be made to define zebrafish stage-specific nutritional requirements and develop a standardized artificial sterile diet to bring GF zebrafish to adulthood. Meanwhile, maintaining water quality without denitrifying bacteria is also challenging for researchers when zebrafish are raised in a sterile environment. (2) Albeit the techniques are efficient and easy to generate a germ-free zebrafish model, it is technically difficult to raise GF zebrafish to adulthood, leading to the research gap of gut microbiota study in GF adult zebrafish. (3) The composition of intestinal microbes in laboratory-reared zebrafish varies greatly among different lab facilities (Stephens et al. 2016; Roeselers et al. 2011), as in the case of mammals (Wu and Wu 2012). In addition, zebrafish exhibit key differences in the core microbiota compared to humans. (4) Environmental conditions and exposures are different in zebrafish compared to humans and other model organisms (Ericsson 2019). (5) Owing to the higher oxygen concentration in the zebrafish intestine, it is another limitation to colonize zebrafish with strictly anaerobic bacteria from human-derived samples (Lu et al. 2021). (6) Though the zebrafish intestine is highly homologous with that of mammals in its development, organization, and function, there are still differences in anatomy and physiology, such as lacking a stomach, crypts of Lieberkühn, and the defensin-secreting Paneth cells (Wallace and Pack 2003; Flores et al. 2020). Hence, we must consider these limitations when extrapolating the findings of gut microbiota in zebrafish to mammals.

## Conclusion

In the past years, the knowledge of the human gut microbiome has expanded rapidly. A range of human diseases is now known to be linked to dysbiosis in gut microbiota. Thus, it is vital to validate the physio- or pathological functions of changed intestinal microbes using experimental animal models. Zebrafish have been a well-used vertebrate model in cellular microbiology for decades. It lends itself to questions that cannot be easily addressed in other model systems, especially the high-resolution in vivo imaging of resident microorganisms. Humans and zebrafish share many similarities in the immune system, nervous system, nutrient metabolism, and cell differentiation or proliferation. It is believed that fully exploring the potential of zebrafish in terms of gut microbiota will provide more therapies for diseases, such as diabetes mellitus, intestinal disease, and neurodegenerative diseases, to maintain gut ecological homeostasis and host health. Although progress has been made, challenges, as mentioned above, remain to be handled in the application of the zebrafish model to gut microbiota studies. In the future, the combination of high-resolution in vivo visualization at the living level with established microbial manipulations in zebrafish will bring new insights into the network of bidirectional communication between gut microorganisms and their host by gut-brain axis, gut-lung axis, or gut-liver axis, which will lead to a better understanding of innovative therapeutic approaches against diseases.

## Data Availability

Not applicable.

## Abbreviations

Apoa1: Apolipoprotein 1
Apoa4: Apolipoprotein 4
ApoB: Apolipoprotein B
Aq3p: Aquaporin 3
Angptl4: Angiopoietin-like 4
BefA: β Cell expansion factor A
Cfl1: Cofilin1
BaP: Benzo[a]pyrene
DBPs: Disinfection by-products
dpf: Day post fertilization
PBDEs: Polybrominated diphenyl ethers
Fdps: Farnesyl-diphosphate synthetase
Fabp2: Fatty acid binding protein 2
Fxr: Farnesoid X receptor
GF: Germ-free
Hpf: Hours post-fertilization
GnRH: Gonadotropin-releasing Hormone
IECs: Intestinal epithelial cells
IGF: Insulin-like growth factor
Irf8: Interferon regulatory factor 8
LSFM: Light sheet fluorescence microscopy
Myd88: Myeloid differentiation primary response 88
NF-кB: Nuclear factor kappa light chain enhancer of activated B cells
PRR: Pattern recognition receptors
Rag1: Recombination activating 1
Saa: Serum amyloid A
SCFAs: Short-chain fatty acids
TNBS: 2,4,6-Trinitrobenzene sulfonic acid
TLRs: Toll-like receptors
Vil1: Villin
